# Association between older people’s health literacy and sociodemographic aspects, functioning, happiness, and perception of the COVID-19 pandemic: a preliminary study

**DOI:** 10.1590/2317-1782/e20240082en

**Published:** 2025-02-10

**Authors:** Beatriz Martins Aragão, Andrezza Gonzalez Escarce, Carla Salles Chamouton, Stela Maris Aguiar Lemos

**Affiliations:** 1 Departamento de Fonoaudiologia, Universidade Federal de Minas Gerais – UFMG - Belo Horizonte (MG), Brasil.; 2 Departamento de Fonoaudiologia, Universidade Estadual Paulista “Júlio de Mesquita Filho” – UNESP - Marília (SP), Brasil.

**Keywords:** Elderly, Happiness, Quality of Life, Health-related Quality of Life, International Classification of Functioning, Disability and Health, Health literacy

## Abstract

**Purpose:**

To analyze the association between older people’s health literacy and sociodemographic aspects, functioning, happiness, and perception of the COVID-19 pandemic.

**Methods:**

This is a preliminary, cross-sectional, analytical, observational study with 37 older adults of both sexes. It used the Brazilian Economic Classification Criteria, WHODAS 2.0, SAHLPA-18, WHO-5 Well-Being Index, and Subjective Happiness Scale, estimated the perception of the pandemic through a questionnaire developed by the authors, and performed descriptive analysis, association analysis using the Pearson chi-square test, and Spearman correlation.

**Results:**

Most participants had inadequate functional health literacy (FHL) results. A higher percentage of individuals from social classes C and D-E had inadequate FHL. The low education level was associated with an inadequate FHL. Most participants with adequate FHL reported feeling “calm and relaxed” for more than half the time or all the time. The SAHLPA-18 score was weakly negatively correlated with the Cognition and Self-Care domains of WHODAS 2.0, indicating that better health literacy is associated with better cognitive and self-care conditions.

**Conclusion:**

Older people with better health literacy had better cognitive ability, better self-care management, higher education levels, and better quality of life.

## INTRODUCTION

Aging causes changes in the body due to the process of senescence and senility, impacting older people’s functional abilities^(1)^.

In 2020, the United Nations proclaimed the “Decade for Healthy Aging in the Americas” (2021-2030), a movement aimed at improving the lives of older individuals, their families, and the communities where they live^(2)^. However, this requires access to healthcare services, focusing on real needs, and recognizing the current flaws in the healthcare system, including communication between professionals and users^(3)^.

Functional health literacy (FHL) is the ability to search for, understand, and share basic health information, and make informed decisions to maintain health^(4)^. This is an important indicator in the older population, as their health literacy levels are often lower than those of the general population^(5)^. Lack of health knowledge and skills can pose barriers to adopting healthy behaviors, impacting functional aspects and quality of life^(1,3,6)^.

Aspects such as health conditions, physical inactivity, lack of family support, and social isolation can influence older people’s quality of life^(7)^. These factors are directly related to their frailty, which may serve as risk indicators for functional disability^(1)^.

Functional aspects of autonomy, independence, quality of life, social-family conditions, spirituality, and work ability in aging may be related to the perception of happiness^(8)^. The latter can be difficult to measure, as it is broadly associated with similar terms such as joy, contentment, satisfaction, and well-being, posing challenges to defining it^(9)^.

The older population was particularly affected by the COVID-19 pandemic, as it was considered a high-risk group from the beginning, with social isolation being one of the recommended sanitary measures. It had significant consequences, such as anxiety, cognitive decline, reduced energy and motivation, and increased sensitivity to threats^(10)^. The authors of a study on this population’s mental health during the COVID-19 pandemic noted that older individuals had greater resilience than younger ones when challenged with social interaction restrictions – a relevant factor for maintaining quality of life^(11)^.

Hence, this study aimed to contribute to the discussion on the importance of FHL and its relationship with functioning, social determinants, quality of life, and the perception of happiness in elderly individuals, enabling a better understanding of the peculiarities related to aging. Thus, the study's objective was to analyze the association between older people’s health literacy and sociodemographic data, functioning, happiness, and perception of the COVID-19 pandemic.

## METHODS

The study was approved by the Research Ethics Committee of the Federal University of Minas Gerais under evaluation report number 4.865.042; CAAE 48816921.6.0000.5149.

This is a preliminary, cross-sectional, analytical, observational study with a non-probabilistic, convenience sample of older adults of both sexes, residing in the urban and rural areas of Itaguara, Minas Gerais, Brazil. After approval from the municipal health department, the health centers’ managers were contacted to select and recruit participants through the Family Health Teams.

The research was presented to the teams, who passed the information on to older adults within their coverage areas. Community health workers scheduled home visits to those interested in the research for data collection with the lead researcher. Thus, 37 participants were recruited for in-person interviews.

The inclusion criteria were being 60 years or older, residing in the municipality selected for the study, and signing an informed consent form.

According to data from the 2022 Brazilian Census^(12)^, Itaguara has an area of 410.468 km^2^ and a population of 13,846 inhabitants – of whom, 2,703 are 60 years or older, corresponding to 19.5% of the population^(12)^. The municipality’s Human Development Index (HDI) is 0.691, placing it in the medium range (HDI 0.600 to 0.699)^(12)^. Longevity is the variable that contributes the most to the municipality's HDI, with an index of 0.836, followed by income and education, with respective scores of 0.697 and 0.567. According to 2021 data from the Brazilian Institute of Geography and Statistics (IBGE), the municipality registered a GDP of R$ 29,790.11 per capita^(12)^.

The following instruments were used for sample characterization:

- 2018 Brazilian Economic Classification Criteria (CCEB)^(13)^, which measures the household’s consumption capacity and investigates the householder’s education level. The scoring guidelines indicate results in classes A, B1, B2, C1, C2, D-E. This study grouped the classes into A-B, C, and D-E for analysis.
- WHO Disability Assessment Schedule (WHODAS 2.0)^(14,15)^, which was developed according to the principles of the biopsychosocial model, providing the functioning level in six life domains (Chart 1).

**Chart 1:** Domains and aspects assessed by WHODAS 2.0

**Domain**	**1. Cognition**	**2. Mobility**	**3. Self-Care**	**4. Getting Along**	**5. Life Activities**	**6. Social Participation**
**Aspects assessed**	Concentration, memory, problem-solving, learning, and communication skills.	Activities such as standing, moving around indoors, staying outdoors, and walking long distances.	Personal care such as hygiene, dressing, eating, and being alone.	Interactions with others and challenges posed by a health condition.	Difficulties with most days’ activities, including household tasks, leisure, work, and school.	Social aspects, such as participation in community activities, challenges and obstacles in the person's environment, and concerns related to preserving personal dignity.

The research used the complete versions in the interviewer-administered and proxy modes, each with 36 questions. The respondent to the latter version is the caregiver of the older person participating in the study. The instrument assesses the domains of cognition, mobility, self-care, getting along, life activities (difficulties in daily routines), and social participation on a scale ranging from (1) no difficulty to (5) extreme difficulty/incapable. At the end, the instrument provides a final score ranging from 0 to 100 (from better to worse). This study used the simple scoring method, in which the scores assigned to each item are summed to find the score for each domain.

- Short Assessment of Health Literacy for Portuguese-Speaking Adults (SAHLPA-18)^(16)^, which assesses the pronunciation and comprehension skills of common medical terms, estimating adults’ health literacy level. The instrument's score ranges from 0 to 18 – scores between 0 and 14 points suggest inadequate health literacy, while those above 14 points indicate adequate health literacy.
- WHO-5 Well-Being Index (WHO-5)^(17)^, a 5-item questionnaire with responses on a Likert scale ranging from 0 to 5, where 0 indicates “at no time” and 5 indicates “all of the time”. The questions refer to how the person felt during the 2 weeks prior to the test administration. The total score ranges from 0 to 25, where 0 represents the worst and 25 represents the best well-being index. The score can also be transformed into percentages (0 to 100%).
- Subjective Happiness Scale^(18)^, which measures the person's sense of happiness. The person must indicate on an ascending scale from 1 to 7 the score that best represents them, where 1 indicates the lowest, and 7 indicates the highest level of happiness. To calculate the result, the score for question 4, which measures unhappiness, must be reversed, then added to the other scores and divided by four. The resulting value indicates the current level of subjective happiness.
- Perception of the COVID-19 Pandemic, a questionnaire the researchers developed to understand older people's perceptions of the COVID-19 pandemic. The instrument has eight questions, although this study addressed only one, “How did you feel during the pandemic?”. It was assessed using a visual analog scale indicating a) – very happy; b) – happy; c) – indifferent; d) – sad; and e) – very sad.

The explanatory variables in this study were participant characterization (sex, age, years of education, and economic classification), WHO-5 (WHO Well-Being Index – Five), WHODAS 2.0, Perception of the COVID-19 Pandemic, and the Subjective Happiness Scale. The response variable was the health literacy index according to the SAHLPA-18.

Descriptive data analysis was performed through the frequency distribution of categorical variables and analysis of measures of central tendency and dispersion for continuous variables. Pearson's chi-square test was used for association analysis, with a 5% significance level. Correlation analysis was also performed with Spearman's correlation coefficient, measuring the magnitude of the correlation as follows: weak = 0.0-0.4; moderate = 0.4-0.7; and strong = 0.7-1.0, provided that the p-value was ≤ 0.05^(19)^. The software used was SPSS, version 25.0.

## RESULTS

The total sample had 37 participants, most of whom were females (75.6%), aged 70 to 79 years (43.2%), followed by those aged 60 to 69 years (40.5%) and over 80 years (16.3%). Their mean age was 72.8 years, with a median of 71 years (SD = 7.74). Most participants had less than 4 years of schooling (81.0%), and the majority (46%) belonged to economic class C, with a mean income ranging from R$ 1,691.44 to R$ 2,965.69.

Most participants (56.8%) had inadequate SAHLPA-18 results. The mean WHO-5 score was 18.46, with a median of 21 points (SD = 6.66), indicating that this population has a better quality of life.

The WHODAS 2.0 findings were as follows:

- The Cognition domain had a mean of 7.97 and a median of 7 points (SD = 2.86), considering that a score of 6 indicates no difficulty in performing the activities in the questions, and a score of 30 corresponds to extreme difficulty or inability to perform the activities.
- The Mobility domain had a mean of 8.14 and a median of 6 points (SD = 4.44), considering that a score of 5 indicates no difficulty in performing the activities in the questions, and a score of 25 corresponds to extreme difficulty or inability to perform the activities.
- The Self-care domain had a mean of 4.54 and a median of 4 points (SD = 1.52), considering that a score of 4 indicates no difficulty in performing the activities in the questions, and a score of 20 corresponds to extreme difficulty or inability to perform the activities.
- The Getting Along domain had a mean of 6.11 and a median of 5 points (SD = 1.96), considering that a score of 5 indicates no difficulty in performing the activities in the questions, and a score of 25 corresponds to extreme difficulty or inability to perform the activities.
- The Life Activities domain had a mean of 6.38 and a median of 5 points (SD = 3.93), considering that a score of 4 indicates no difficulty in performing the activities in the questions, and a score of 20 corresponds to extreme difficulty or inability to perform the activities.
- The Participation domain had a mean of 10.57 and a median of 8 points (SD = 4.75), considering that a score of 8 indicates no difficulty in performing the activities in the questions, and a score of 40 corresponds to extreme difficulty or inability to perform the activities.

The mean final happiness level in the Subjective Happiness Scale was 5.89, with a median of 5.75 (SD = 3.21), indicating a good perception of happiness among the participating older adults.

The association analysis between the SAHLPA-18 and sociodemographic data found a statistically significant result between years of schooling and the SAHLPA-18 score (p = 0.012). On the other hand, SAHLPA-18 was not statistically significantly associated with sex, age, or socioeconomic aspects (p > 0.05) (Table 1).

**Table 1:** Association between SAHLPA-18 and sociodemographic data

**Variables**		**SAHLPA**		**p-value**
		**Adequate N (%)**	**Inadequate N (%)**	
**Sex**				
	Females	13(81.3)	15(71.4)	0.490
	Males	3(18.8)	6(28.6)	
	Total	16(100)	21(100)	
**CCEB**				
	A-B	7(43.8)	3(14.3)	0.131
	C	6(37.5)	11(52.4)	
	D-E	3(18.8)	7(33.3)	
	Total	16(100)	21(100)	
**Age**				
	60-69 years	7(43.8)	8(38.1)	0.819
	70-79 years	6(37.5)	10(47.6)	
	Over 80 years	3(18.8)	3(14.3)	
	Total	16(100)	21(100)	
**Education in years**				
	Less than 4 years	10(62.5)	20(95.2)	0.012*
	More than 4 years	6(37.5)	1(4.8)	
	Total	16(100)	21(100)	

Pearson chi-square test

* p-value ≤ 0.05

**Caption:** N = number of individuals; SAHLPA-18 = Short Assessment of Health Literacy for Portuguese-Speaking Adults

The association analysis between the SAHLPA-18 and WHO-5 found a statistically significant result between FHL and “I felt calm/relaxed during the last 2 weeks” (p = 0.008) (Table 2).

**Table 2:** Association between SAHLPA-18 and WHO-5

**Variables**		**SAHLPA**		**p-value**
		**Adequate N (%)**	**Inadequate N (%)**	
**Cheerful in good spirits**				
	At no time	2(12.5)	0(0.0)	0.175
	Some of the time/Less than half the time	2(12.5)	7(33.3)	
	Most of the time/More than half the time	7(43.8)	6(28.6)	
	All of the time	5(31.3)	8(38.1)	
	Total	16(100)	21(100)	
**Calm and relaxed**				
	At no time	2(12.5)	0(0.0)	0.008*
	Some of the time/Less than half the time	0(0.0)	6(28.6)	
	Most of the time/More than half the time	8(50.0)	3(14.3)	
	All of the time	6(37.5)	12(57.1)	
	Total	16(100)	21(100)	
**Active and vigorous**				
	At no time	2(12.5)	0(0.0)	0.270
	Some of the time/Less than half the time	2(12.5)	6(28.6)	
	Most of the time/More than half the time	5(31.3)	5(23.8)	
	All of the time	7(43.8)	10(47.6)	
	Total	16(100)	21(100)	
**Fresh and rested**				
	At no time	3(18.8)	0(0.0)	0.141
	Some of the time/Less than half the time	3(18.8)	5(23.8)	
	Most of the time/More than half the time	3(18.8)	2(9.5)	
	All of the time	7(43.8)	14(66.7)	
	Total	16(100)	21(100)	
**My daily life has been filled with things that interest me.**				
	At no time	1(6.3)	0(0.0)	0.554
	Some of the time/Less than half the time	1(6.3)	3(14.3)	
	Most of the time/More than half the time	5(31.3)	5(23.8)	
	All of the time	9(56.3)	13(61.9)	
	Total	16(100)	21(100)	

Pearson chi-square test

* p-value ≤ 0.05

**Caption:** N = number of individuals; SAHLPA-18 = Short Assessment of Health Literacy for Portuguese-Speaking Adults; WHO-5 = World Health Organization’s 5-item well-being index

The association between the SAHLPA-18 and the COVID-19 Perception questionnaire found no statistically significant results (Table 3).

**Table 3:** Association between SAHLPA-18 and perception of the COVID-19 pandemic

**Variables**		**SAHLPA**		**p-value**
		**Adequate N (%)**	**Inadequate N (%)**	
**How did you learn about the pandemic?**				
	Family	0(0.0)	3(14.3)	0.209
	Friends	0(0.0)	1(4.8)	
	News	15(93.8)	17(81.0)	
	Healthcare center	1(6.3)	0(0.0)	
	Total	16(100)	21(100)	
**Were there any differences in your routine during the pandemic?**				
	Yes	13(81.3)	14(66.7)	0.322
	No	3(18.8)	7(33.3)	
	Total	16(100)	21(100)	
**How did you feel during the pandemic?**				
	Very happy	1(6.3)	1(4.8)	0.671
	Happy	2(12.5)	3(14.3)	
	Normal	6(37.5)	4(19.0)	
	Sad	4(25.0)	5(23.8)	
	Very sad	3(18.8)	8(38.1)	
	Total	16(100)	21(100)	
**Do you know what social isolation is?**				
	Yes	16(100)	17(81.0)	0.065
	No	0(0.0)	4(19.0)	
	Total	16(100)	21(100)	
**Was there isolation in your city?**				
	Yes	10(62.5)	18(85.7)	0.176
	No	2(12.5)	2(9.5)	
	Partially	4(25.0)	1(4.8)	
	Total	16(100)	21(100)	
**Where did you spend the isolation period?**				
	At home	12(75.0)	15(71.4)	0.923
	At a relative’s home	1(6.3)	1(4.8)	
	Partly at home and partly at a relative's home	3(18.8)	5(23.8)	
	Total	16(100)	21(100)	
**Did you stop doing any pleasurable activities during the isolation period?**				
	Yes	11(68.8)	14(66.7)	0.893
	No	5(31.3)	7(33.3)	
	Total	16(100)	21(100)	
**Did you feel lonely during isolation?**				
	Yes	6(37.5)	12(57.1)	0.236
	No	10(62.5)	9(42.9)	
	Total	16(100)	21(100)	
			

Pearson chi-square test

**Caption:** N = number of individuals; SAHLPA-18 = Short Assessment of Health Literacy for Portuguese-Speaking Adults

The correlation analysis between the SAHLPA-18 (raw score) and age, WHODAS 2.0 test, and total Subjective Happiness Scale score revealed a weak negative correlation with statistical significance between the SAHLPA-18 score and the WHODAS 2.0 Cognition (-0.351) and Self-Care (0.337) domains. This indicates that the higher the SAHLPA-18 score, the lower the scores in the Cognition and Self-Care domains (Table 4).

**Table 4:** Correlation between age, WHODAS 2.0 domains, Subjective Happiness Scale, and SAHLPA-18

**Variables**	**SAHLPA**
**Age**	-0.182
**WHODAS – Cognition**	-0.351*
**WHODAS – Mobility**	-0.166
**WHODAS – Self-Care**	-0.337*
**WHODAS – Getting Along**	-0.102
**WHODAS – Life Activities**	-0.184
**WHODAS – Social Participation**	0.227
**Happiness**	0.243
	

Spearman Correlation Coefficient

* p-value ≤ 0.05

**Caption:** WHODAS 2.0 = World Health Organization’s Disability Assessment Schedule; SAHLPA-18 = Short Assessment of Health Literacy for Portuguese-Speaking Adults

## DISCUSSION

Inadequate FHL was prevalent in more than half of the study participants, which is in line with the literature^(3,5)^. Although age was not statistically significantly associated with an inadequate SAHLPA-18, some studies indicate a decrease in FHL performance with increasing age^(3,5,20)^.

Moreover, the percentage of people from Classes C and D-E was higher among subjects with inadequate FHL, although no statistically significant association was found. The literature points to a relationship between socioeconomic factors and health literacy, where those with lower health literacy tend to be at higher risk of social vulnerability^(4)^.

The care process must consider these aspects because, according to the World Health Organization, they are social determinants of health^(21)^, making FHL a factor influencing health problems and their risk factors.

The statistical significance between education and inadequate FHL corroborates the national literature^(3,5,20)^, which states that older adults with an inadequate FHL also have low education levels. This data is important, as it can influence this population’s ability to obtain and use health information^(3,5,20)^.

It also highlights that a person with basic literacy is not guaranteed the competence to fully read and understand all types of written texts and various concepts. Considering FHL as a multidimensional set of competencies and skills, it is assumed to be a complex chain of constructs requiring basic reading, writing, mathematics, communication skills, recognizing risks, having critical thinking to analyze conflicting information, and making health-related decisions to be considered when planning and carrying out health actions^(22,23)^.

The association analysis in this study revealed statistical significance between the SAHLPA-18 and WHO-5’s “calm and relaxed” since most participants with an adequate FHL reported feeling so more than half of the time or all the time. This data should be analyzed in light of the importance of FHL for maintaining quality of life. The literature shows that well-being and quality of life are linked to the person’s family relationships, friendships, social support, and degree of functional dependence^(24,25)^.

The absence of statistical significance between the perception of the COVID-19 pandemic and the SAHLPA-18 demonstrates that the level of FHL did not influence the perception of the pandemic in the study sample. Although some studies have shown that the distancing and protective measures adopted during the pandemic impacted the mental health of part of the older population^(10,11,26)^, the literature also indicates that older adults had better mechanisms to cope with the period of isolation than younger individuals^(10,11,27)^.

Coping with moments of tension and uncertainty, such as during the COVID-19 pandemic, requires psychosocial resources and resilient strategies to fight crises. Older adults achieved it through organizing daily routines at home and within the community, improving lifestyle habits – including physical activity – and fostering positive behavior, all aimed at maintaining well-being and providing social support to others^(10,11,26,27)^.

The results also indicate that the SAHLPA-18 score was weakly negatively correlated with the WHODAS 2.0 Cognition and Self-Care domains. The WHODAS scoring is inverse, meaning that lower scores indicate better functional clinical status. This finding aligns with the literature, which suggests that higher health literacy is associated with better cognitive conditions and self-care abilities^(5,20)^.

It is important to highlight the role of caregivers and family members for older adults with cognitive impairments, not only in facilitating care management but also in evaluating and using health information. Just as the users’ health literacy is relevant for effective care, that of caregivers also significantly impacts these individuals’ quality of life^(28)^.

Studies indicate that a lack of guidance and support for caregivers leads to stress and overload, especially when they care for individuals with higher levels of dependency. Combined with caregivers’ lack of self-care practices, their health literacy condition significantly influences the health outcomes of those under their care. Challenges such as difficulty understanding and interacting with healthcare professionals, insufficient information, or difficulty evaluating the quality of information are examples of the consequences of inadequate health literacy among caregivers, which can affect their ability to provide effective care^(28,29)^.

As limitations of the present study, it is important to note that, being a preliminary stage of an observational study, the sample size and design do not allow for generalizations. Furthermore, the questions related to the perception of the COVID-19 pandemic may have a restricted scope.

Nevertheless, developing a preliminary study allowed for assessing the feasibility of research with a larger sample, stratified by sex, age, and functional aspects, to obtain more robust evidence.

A key point of this study was the understanding that health literacy encompasses multiple dimensions and that triangulation with functioning, quality of life, and perception of happiness proves to be appropriate. Furthermore, analyzing the associations of health literacy with the perception of the pandemic may contribute to discussions about decision-making in health practices, interventions, and adherence to health interventions among the older population.

## CONCLUSION

The analysis of the association between health literacy, sociodemographic aspects, functioning, quality of life, happiness, and older people's perception of the pandemic revealed a relationship between health literacy and education level, cognition, self-care, and feeling calm and relaxed.

Therefore, it can be stated that older adults in the study sample with better health literacy also had higher cognitive skills and education levels, and better self-care management and well-being outcomes.
