# Supplementary material for: Association between older people’s health literacy and sociodemographic aspects, functioning, happiness, and perception of the COVID-19 pandemic: a preliminary study
Source: Codas. 2025 Feb 10;37(2):e20240082. doi: 10.1590/2317-1782/e20240082en (PMC11813177; doi:10.1590/2317-1782/e20240082en)
Supplement: Translation

# Translation: Associação entre letramento em saúde e aspectos sociodemográficos, funcionalidade, felicidade e percepção da pandemia de COVID-19 de pessoas idosas: estudo preliminar

**DOI:** 10.1590/2317-1782/e20240082pt

**Authors:** Beatriz Martins Aragão, Andrezza Gonzalez Escarce, Carla Salles Chamouton, Stela Maris Aguiar Lemos

**Affiliations:** 1 Departamento de Fonoaudiologia, Universidade Federal de Minas Gerais – UFMG - Belo Horizonte (MG), Brasil.; 2 Departamento de Fonoaudiologia, Universidade Estadual Paulista “Júlio de Mesquita Filho” – UNESP - Marília (SP), Brasil.

**Keywords:** Idoso, Felicidade, Qualidade de Vida, Qualidade de Vida Relacionada à Saúde, Classificação Internacional de Funcionalidade, Incapacidade e Saúde, Letramento em Saúde

## Abstract

**Objetivo:**

Analisar a associação entre o Letramento em saúde de pessoas idosas e funcionalidade, felicidade, percepção da pandemia de Covid-19 e aspectos sociodemográficos.

**Método**

Estudo preliminar com delineamento observacional analítico transversal com amostra de 37 pessoas idosas, de ambos os gêneros. Os instrumentos utilizados foram o Critério de Classificação Econômica Brasil, WHODAS 2.0, SAHLPA-18, Índice de Bem-Estar OMS - 5, Escala de Felicidade Subjetiva e a percepção da pandemia foi estimada com um questionário desenvolvido pelas autoras. Foram realizadas análises descritivas, de associação, por meio do Teste Qui-quadrado de Pearson, e de correlação de Spearman.

**Resultados:**

A maioria dos participantes apresentou resultado inadequado no Letramento Funcional em Saúde (LFS). A porcentagem de pessoas das classes C e D-E foi maior entre as que apresentaram LFS inadequado. Houve associação entre baixo nível de escolaridade e LFS inadequado. A maioria dos participantes com LFS adequado respondeu permanecer mais da metade do tempo ou o tempo todo “calmo(a) e tranquilo(a)”. Foi observado correlação negativa de magnitude fraca entre o escore do SAHLPA-18 e os domínios Cognição e Autocuidado WHODAS 2.0, que indica, quanto melhor o letramento em saúde melhores são as condições cognitivas e de autocuidado.

**Conclusão:**

As pessoas idosas que apresentaram melhor letramento em saúde demonstraram melhor capacidade cognitiva, melhor gestão do autocuidado, maior grau de instrução e melhor qualidade de vida.

## INTRODUÇÃO

Com o avanço da idade, ocorrem mudanças no organismo decorrentes do processo de senescência e senilidade, que podem impactar nas capacidades funcionais das pessoas idosas^(1)^.

No ano de 2020, foi proclamada, pelas Nações Unidas, a “Década para o Envelhecimento Saudável das Américas” (2021-2030), um movimento que tem como objetivo melhorar a vida de pessoas idosas, suas famílias e a comunidade em que vivem^(2)^. No entanto, para que isso ocorra é necessária a garantia de acesso aos serviços de saúde, com foco nas necessidades reais e reconhecendo as falhas que ocorrem no sistema assistencial, até mesmo na comunicação entre profissional e usuário^(3)^.

A capacidade de busca, compreensão e compartilhamento de informações básicas sobre saúde e de tomada de decisões acertadas para manutenção desta, definem o conceito de alfabetismo ou letramento funcional em saúde^(4)^. Na população idosa este é um indicador importante, pois os níveis de letramento em saúde dessas pessoas costumam ser menores do que na população em geral^(5)^. A falta de conhecimento e as habilidades acerca da própria saúde podem ser considerados barreiras para a adesão de comportamentos saudáveis, o que pode impactar nos aspectos funcionais e na qualidade de vida^(1,3,6)^.

Aspectos como condições de saúde, inatividade física, insuficiência familiar e isolamento social podem influenciar a qualidade de vida da pessoa idosa^(7)^, além de serem fatores diretamente relacionados à fragilidade dessa população, podendo ser indicadores de risco para incapacidade funcional^(1)^.

No envelhecimento, os aspectos funcionais de autonomia e independência, a qualidade de vida, as condições sociofamiliares, a espiritualidade, e a capacidade laboral podem estar relacionadas à percepção de felicidade^(8)^. Como conceito, a felicidade pode ser de difícil mensuração, pois em sua amplitude está associada com termos similares como alegria, contentamento, satisfação e bem-estar, o que indica dificuldades para defini-la^(9)^.

Com a pandemia de COVID-19, a população idosa foi particularmente afetada por ser considerada grupo de risco desde o início, tendo como uma das medidas sanitárias recomendadas, o isolamento social. Este último, apresentou consequências importantes, como ansiedade, declínio cognitivo, redução de ânimo e motivação, e maior sensibilidade a ameaças^(10)^. Em estudo realizado sobre a saúde mental desta população durante o período da pandemia de COVID-19, os autores pontuam que, frente aos desafios do impedimento do convívio social, pessoas idosas apresentaram maior resiliência quando comparadas aos mais jovens, sendo um fator relevante para a manutenção da qualidade de vida^(11)^.

Nessa perspectiva, o presente estudo pretende contribuir para a discussão da importância do letramento funcional em saúde e sua relação com a funcionalidade, os determinantes sociais, a qualidade de vida e a percepção da felicidade em pessoas idosas, possibilitando melhor entendimento das peculiaridades relacionadas ao envelhecimento. Deste modo, o objetivo do estudo foi analisar a associação do Letramento em saúde de pessoas idosas segundo funcionalidade, felicidade, percepção da pandemia de Covid-19 e dados sociodemográficos.

## MÉTODO

Este estudo foi aprovado pelo Comitê de Ética em Pesquisa (COEP) da Universidade Federal de Minas Gerais sob o parecer 4.865.042; CAAE 48816921.6.0000.5149.

Trata-se de estudo preliminar com delineamento observacional analítico transversal com amostra não probabilística e por conveniência composta por pessoas idosas, de ambos os sexos, residentes das zonas urbana e rural do município de Itaguara-MG. Após anuência da secretaria municipal de saúde, foi realizado contato com os gestores dos centros de saúde, para seleção e recrutamento dos participantes via Equipes de Saúde da Família.

A pesquisa foi divulgada para as equipes, que repassaram as informações às pessoas idosas das áreas de abrangência, e os que tinham interesse, tinham visitas domiciliares agendadas pelos Agentes Comunitários de Saúde acompanhados pela pesquisadora responsável pela coleta de dados. Assim, foram recrutados 37 participantes para entrevista presencial.

Foram adotados como critérios de inclusão: ter 60 anos ou mais, residir no município selecionado para o estudo e assinar o Termo de Consentimento Livre e Esclarecido.

De acordo com dados do Censo de 2022^(12)^, Itaguara dispõe de uma área de 410.468 km^2^ e uma população de 13.846 habitantes. Destes, 2.703 apresentam 60 anos ou mais, o que corresponde a 19,5% da população^(12)^. O Índice de Desenvolvimento Humano (IDHM) do município é 0,691, o que o situa em uma faixa de Desenvolvimento Humano Médio (IDHM 0,600 a 0,699)^(12)^. A variável que mais contribui para o IDHM da cidade é a Longevidade com índice de 0,836, seguida da Renda e Educação com 0,697 e 0,567, respectivamente. Segundo dados do IBGE, em 2021, o município registrou PIB per capita de R$ 29.790,11^(12)^.

Para caracterização da amostra, foram utilizados os seguintes instrumentos:

- Critério de Classificação Econômica Brasil (CCEB 2018)^(13)^ – tem como objetivo mensurar a capacidade de consumo de um domicílio e investigar o grau de escolaridade do chefe da família. Conforme normatização da pontuação obtida, apresenta seu resultado em classes, a saber: A, B1, B2, C1, C2, D-E. Para análise do presente estudo, as classes foram agrupadas em A-B, C e D-E.
  -WHO Disability Assessment Schedule (WHODAS 2.0)^(14,15)^ – instrumento elaborado segundo as premissas do modelo biopsicossocial que fornece o nível de funcionalidade em seis domínios da vida (Quadro 1).

**Quadro 1:** Apresentação dos domínios e aspectos avaliados pelo WHODAS 2.0

**Domínio**	**1. Cognição**	**2. Mobilidade**	**3. Autocuidado**	**4. Relacionamento**	**5. Atividades de vida**	**6. Participação**
**Aspectos avaliados**	Concentração, memória, resolução de problemas, aprendizado e habilidades comunicativas.	Atividades como ficar em pé, movimentar-se dentro de casa, permanecer fora de casa e caminhar longas distâncias.	Cuidados pessoais como higiene, vestir-se, alimentar-se e ficar sozinho.	Interações com outras pessoas e os desafios enfrentados devido a uma condição de saúde.	Dificuldades com as atividades realizadas na maioria dos dias, incluindo tarefas relacionadas às responsabilidades domésticas, lazer, trabalho e escola.	Aspetos sociais, como participação em atividades comunitárias, desafios e obstáculos no ambiente da pessoa, e preocupações relacionadas à preservação da dignidade pessoal.

Na pesquisa, foram contempladas as versões completas, compostas por 36 questões cada, nos modos “aplicada por entrevistador” e proxy, nesta última versão o respondente é o cuidador da pessoa idosa participante do estudo. O instrumento inclui a avaliação dos seguintes domínios: cognição; mobilidade; autocuidado; relacionamento; atividades de vida (dificuldades na rotina diária); participação nas dimensões sociais. Os domínios são avaliados em uma escala que varia de (1) sem dificuldade a (5) extrema dificuldade/incapaz. Ao final o instrumento apresenta um escore final variando de 0 a 100 (melhor para pior). Neste estudo foi adotado o método de pontuação simples, no qual as pontuações atribuídas a cada um dos itens são somadas para encontrar a pontuação referente a cada domínio.

- Short Assessment of Health Literacy for Portuguese-Speaking Adults (SAHLPA-18)^(16)^ – avalia as habilidades de pronúncia e compreensão de termos médicos comuns, sendo utilizado para estimar o nível de alfabetismo/letramento em saúde de adultos. A pontuação do instrumento varia entre 0 e 18. O escore entre 0 e 14 pontos sugere alfabetismo em saúde inadequado e acima de 14 pontos alfabetismo adequado.
- Índice de Bem-Estar OMS - 5 (WHO-5)^(17)^ – questionário composto por cinco questões, com resposta disposta em escala Likert que varia de 0 a 5, na qual o 0 indica “nenhum momento” e 5 a “o tempo todo”. As questões referem-se a como a pessoa se sentiu nas duas semanas anteriores à aplicação do teste. O escore total varia de 0 a 25, em que 0 representa o pior e 25, o melhor índice de bem-estar. O escore também pode ser transformado em valores percentuais (0 a 100%).
- Escala de Felicidade Subjetiva^(18)^ – instrumento que mensura a sensação de felicidade individual. A pessoa deve indicar, em uma escala ascendente de 1 a 7, a pontuação que melhor a representa, em que 1 indica menor nível de felicidade e 7 maior nível. Para calcular o resultado, é necessário inverter a pontuação da questão 4, que trata da medida de infelicidade, e somar às demais pontuações e dividir o número resultante por quatro. O valor obtido indica o nível de felicidade subjetiva atual.
- Percepção da Pandemia da COVID-19 – questionário elaborado pelas pesquisadoras com o objetivo de compreender a percepção das pessoas idosas acerca da pandemia da COVID-19. O roteiro é composto por oito perguntas, no entanto, neste estudo abordamos apenas uma das questões, a saber: “Como você se sentiu durante o período da pandemia?”. A questão foi abordada por meio de escala visual analógica que indicava: a) – muito feliz; b) – feliz; c) – indiferente; d) – triste; e) – muito triste;

Na presente pesquisa, as variáveis explicativas foram: caracterização dos participantes (sexo, idade, escolaridade em anos e classificação econômica), WHO-5 (Índice de Bem-estar da OMS – Cinco), WHODAS 2.0, Percepção da pandemia da COVID-19 e a Escala de Felicidade Subjetiva. Como variável resposta, foi considerado o índice de alfabetismo em saúde segundo o protocolo SAHLPA-18.

A análise descritiva dos dados foi realizada por meio da distribuição de frequência das variáveis categóricas e análise das medidas de tendência central e de dispersão das variáveis contínuas. Já para as de associação, foi utilizado o Teste Qui-quadrado de Pearson com nível de significância de 5%. Também foi realizada análise de correlação, por meio do coeficiente de correlação de Spearman, no qual foi a magnitude da correlação foi medida seguindo o seguinte parâmetro: fraca = 0,0-0,4; moderada = 0,4-0,7 e forte = 0,7-1,0; desde que apresentasse valor de p≤0,05^(19)^. O software utilizado foi o SPSS, versão 25.0.

## RESULTADOS

A amostra total foi composta por 37 participantes, dos quais a maioria era do sexo feminino (75,6%) e a maior parte estava na faixa etária de 70-79 anos (43,2%), seguido dos de 60-69 anos (40,5%) e, por fim, das pessoas idosas com mais de 80 anos (16,3%). A média de idade foi de 72,8 anos com mediana de 71 anos (DP=7,74). A maioria dos participantes apresentou menos que quatro anos de escolaridade (81,0%) e a maior parte (46%) pertencia à classe econômica C, apresentando a média salarial entre R$1.691,44 e R$2.965,69.

No SAHLPA-18, a maioria apresentou resultado inadequado (56,8%). Já no WHO-5 a média da pontuação foi de 18,46 com mediana de 21 pontos (DP=6,66) o que indica que esta população possui uma melhor qualidade de vida.

No instrumento WHODAS 2.0 obteve-se os seguintes achados:

- Domínio Cognição apresentou média de 7,97 e mediana de 7 pontos (DP= 2,86), considerando que a pontuação de 6 equivale a nenhuma dificuldade para realizar as atividades questionadas e 30, corresponde a extrema dificuldade ou que não consegue realizar as atividades.
- Domínio Mobilidade apresentou média de 8,14 e mediana de 6 pontos (DP= 4,44), considerando que a pontuação de 5 equivale a nenhuma dificuldade para realizar as atividades questionadas e 25, corresponde a extrema dificuldade ou que não consegue realizar as atividades.
- Domínio Autocuidado apresentou média de 4,54 e mediana de 4 pontos (DP= 1,52), considerando que a pontuação de 4 equivale a nenhuma dificuldade para realizar as atividades questionadas e 20, corresponde a extrema dificuldade ou que não consegue realizar as atividades.
- Domínio Relações interpessoais apresentou média de 6,11 e mediana de 5 pontos (DP=1,96), considerando que a pontuação de 5 equivale a nenhuma dificuldade para realizar as atividades questionadas e 25, corresponde a extrema dificuldade ou que não consegue realizar as atividades.
- Domínio Atividades domésticas apresentou média de 6,38 e mediana de 5 pontos (DP= 3,93), considerando que a pontuação de 4 equivale a nenhuma dificuldade para realizar as atividades questionadas e 20, corresponde a extrema dificuldade ou que não consegue realizar as atividades.
- Domínio Participação apresentou média de 10,57 e mediana de 8 pontos (DP= 4,75), considerando que a pontuação de 8 equivale a nenhuma dificuldade para realizar as atividades questionadas e 40, corresponde a extrema dificuldade ou que não consegue realizar as atividades.

Em relação à Escala de Felicidade Subjetiva, o nível final de felicidade teve média de 5,89 e mediana de 5,75 (DP = 3,21), o que indica uma boa percepção de felicidade por parte das pessoas idosas.

Na análise de associação entre o SAHLPA-18 e os dados sociodemográficos houve resultado com significância estatística entre escolaridade em anos e resultado do SAHLPA-18 (p=0,012). As demais associações, entre SAHLPA-18 e as variáveis sexo, idade e aspectos socioeconômicos não apresentaram resultado com significância estatística (p>0,05) (Tabela 1).

**Tabela 1:** Associação entre SAHLPA - 18 e Dados Sociodemográficos

**Variáveis**		**SAHLPA**		**p-valor**
		**Adequado N (%)**	**Inadequado N (%)**	
Sexo				
	Feminino	13(81,3)	15(71,4)	0,490
	Masculino	3(18,8)	6(28,6)	
	Total	16(100)	21(100)	
CCEB				
	A-B	7(43,8)	3(14,3)	0,131
	C	6(37,5)	11(52,4)	
	D-E	3(18,8)	7(33,3)	
	Total	16(100)	21(100)	
Idade				
	60-69 anos	7(43,8)	8(38,1)	0,819
	70-79 anos	6(37,5)	10(47,6)	
	Maior que 80 anos	3(18,8)	3(14,3)	
	Total	16(100)	21(100)	
Escolaridade em anos				
	Menos que 4 anos	10(62,5)	20(95,2)	0,012*
	Mais que 4 anos	6(37,5)	1(4,8)	
	Total	16(100)	21(100)	

Teste Qui-quadrado de Pearson

* valor de p≤0,05

**Legenda:** N = número de indivíduos; SAHLPA-18 = Short Assessment of Health Literacy for Portuguese-Speaking Adults

Na análise de associação entre o SAHLPA-18 e o WHO-5, observou-se resultado com significância estatística entre o letramento funcional e saúde e o item “Senti-me calmo/tranquilo(a) durante as últimas duas semanas” (p=0,008) (Tabela 2).

**Tabela 2:** Associação entre SAHLPA-18 e WHO-5

**Variáveis**		**SAHLPA**		**p-valor**
		**Adequado N (%)**	**Inadequado N (%)**	
**Alegre e bem disposto/a**				
	Nunca	2(12,5)	0(0,0)	0,175
	Algumas vezes/menos da metade do tempo	2(12,5)	7(33,3)	
	Maior parte do tempo/ mais da metade do tempo	7(43,8)	6(28,6)	
	Todo o tempo	5(31,3)	8(38,1)	
	Total	16(100)	21(100)	
**Calmo e tranquilo/a**				
	Nunca	2(12,5)	0(0,0)	0,008*
	Algumas vezes/menos da metade do tempo	0(0,0)	6(28,6)	
	Maior parte do tempo/ mais da metade do tempo	8(50,0)	3(14,3)	
	Todo o tempo	6(37,5)	12(57,1)	
	Total	16(100)	21(100)	
**Ativo e Enérgico/a**				
	Nunca	2(12,5)	0(0,0)	0,270
	Algumas vezes/menos da metade do tempo	2(12,5)	6(28,6)	
	Maior parte do tempo/ mais da metade do tempo	5(31,3)	5(23,8)	
	Todo o tempo	7(43,8)	10(47,6)	
	Total	16(100)	21(100)	
**Fresco/a e repousado/a**				
	Nunca	3(18,8)	0(0,0)	0,141
	Algumas vezes/menos da metade do tempo	3(18,8)	5(23,8)	
	Maior parte do tempo/ mais da metade do tempo	3(18,8)	2(9,5)	
	Todo o tempo	7(43,8)	14(66,7)	
	Total	16(100)	21(100)	
**O meu dia a dia tem sido preenchido com coisas que me interessam?**				
	Nunca	1(6,3)	0(0,0)	0,554
	Algumas vezes/menos da metade do tempo	1(6,3)	3(14,3)	
	Maior parte do tempo/ mais da metade do tempo	5(31,3)	5(23,8)	
	Todo o tempo	9(56,3)	13(61,9)	
	Total	16(100)	21(100)	

Teste Qui-quadrado de Pearson

* valor de p≤0,05

**Legenda:** N = número de indivíduos; SAHLPA-18 = Short Assessment of Health Literacy for Portuguese-Speaking Adults; WHO-5 = Índice de Bem-Estar OMS-5

Já na associação entre SAHLPA-18 e o questionário de Percepção da pandemia da COVID-19, não foi observado resultados com significância estatística (Tabela 3).

**Tabela 3:** Associação entre o SAHLPA-18 e Percepção da pandemia da COVID-19

**Variáveis**		**SAHLPA**		**p-valor**
		**Adequado N (%)**	**Inadequado N (%)**	
**Como tomou conhecimento da pandemia**				
	Família	0(0,0)	3(14,3)	0,209
	Amigos	0(0,0)	1(4,8)	
	Noticiários	15(93,8)	17(81,0)	
	Centro de saúde	1(6,3)	0(0,0)	
	Total	16(100)	21(100)	
**Durante o período de pandemia houve alguma diferença em sua rotina**				
	Sim	13(81,3)	14(66,7)	0,322
	Não	3(18,8)	7(33,3)	
	Total	16(100)	21(100)	
**Como se sentiu durante o período da pandemia**				
	Muito feliz	1(6,3)	1(4,8)	0,671
	Feliz	2(12,5)	3(14,3)	
	Normal	6(37,5)	4(19,0)	
	Triste	4(25,0)	5(23,8)	
	Muito triste	3(18,8)	8(38,1)	
	Total	16(100)	21(100)	
**Sabe o que é isolamento social**				
	Sim	16(100)	17(81,0)	0,065
	Não	0(0,0)	4(19,0)	
	Total	16(100)	21(100)	
**Houve isolamento em sua cidade**				
	Sim	10(62,5)	18(85,7)	0,176
	Não	2(12,5)	2(9,5)	
	Parcialmente	4(25,0)	1(4,8)	
	Total	16(100)	21(100)	
**Onde passou o período de isolamento**				
	Em casa	12(75,0)	15(71,4)	0,923
	Casa de familiar	1(6,3)	1(4,8)	
	Parte em casa e parte na casa do familiar	3(18,8)	5(23,8)	
	Total	16(100)	21(100)	
**No período de isolamento deixou de realizar alguma atividade prazerosa**				
	Sim	11(68,8)	14(66,7)	0,893
	Não	5(31,3)	7(33,3)	
	Total	16(100)	21(100)	
**Se sentiu sozinho durante o isolamento**				
	Sim	6(37,5)	12(57,1)	0,236
	Não	10(62,5)	9(42,9)	
	Total	16(100)	21(100)	
			

Teste Qui-quadrado de Pearson

**Legenda:** N = número de indivíduos; SAHLPA-18 = Short Assessment of Health Literacy for Portuguese-Speaking Adults

A análise de correlação entre o SAHLPA-18 (pontuação bruta) e as variáveis idade, teste WHODAS 2.0 e escore total da Escala de Felicidade Subjetiva, revelou correlação negativa de magnitude fraca com significância estatística entre o escore do SAHLPA-18 e os domínios Cognição (-0,351) e Autocuidado (0,337) do WHODAS 2.0. Esse resultado indica que quanto maior o escore do SAHLPA-18, menor os dos Domínios Cognição e Autocuidado (Tabela 4).

**Tabela 4:** Correlação entre as variáveis Idade, Domínios do WHODAS 2.0, Escala de Felicidade Subjetiva e SAHLPA-18

**Variáveis**	**SAHLPA**
**Idade**	-0,182
**WHODAS – Cognição**	-0,351*
**WHODAS – Mobilidade**	-0,166
**WHODAS – Autocuidado**	-0,337*
**WHODAS – Relações Interpessoais**	-0,102
**WHODAS – Atividades domésticas**	-0,184
**WHODAS – Participação**	0,227
**Felicidade**	0,243
	

Coeficiente de Correlação de Spearman

* p-valor ≤ 0,05

**Legenda:** WHODAS 2.0 = WHO Disability Assessment Schedule; SAHLPA-18 = Short Assessment of Health Literacy for Portuguese-Speaking Adults

## DISCUSSÃO

No presente estudo, o Letramento Funcional em Saúde (LFS) inadequado foi prevalente em mais da metade dos participantes, o que está em consonância com a literatura^(3,5)^. Apesar da variável idade não apresentar associação com significância estatística entre aqueles com SAHLPA-18 inadequado, alguns trabalhos indicam que com o aumento da idade há diminuição do desempenho no LFS^(3,5,20)^.

Além disso, a porcentagem de pessoas das Classes C e D-E foi maior entre os sujeitos que apresentaram LFS inadequado, apesar de não ter sido verificada associação com significância estatística. Cabe ressaltar que a literatura aponta relação entre os aspectos socioeconômicos e o letramento em saúde, em que quem apresenta menor letramento em saúde tende a estar em maior risco de vulnerabilidade social^(4)^.

Estes aspectos devem ser considerados para o processo de cuidado, pois segundo a Organização Mundial da Saúde, são determinantes sociais da saúde^(21)^, o que torna o LFS um fator que influencia na ocorrência de problemas de saúde e seus fatores de risco.

A significância estatística presente entre a escolaridade e o LFS inadequado corrobora a literatura nacional^(3,5,20)^ que refere que as pessoas idosas que possuem LFS inadequado também apresentam baixa escolaridade. Tal dado é importante, visto que esse fator pode influenciar na capacidade de obtenção e uso das informações em saúde por essa população^(3,5,20)^.

Este fato evidencia que a pessoa com letramento básico, não tem garantia de competência para ler e compreender todos os tipos de texto escrito e nem entender conceitos de variados em sua totalidade. Considerando o LFS enquanto conjunto multidimensional de competências e habilidades, pressupõe-se uma cadeia complexa de constructos que envolve dominar habilidades de leitura básica, escrita, matemática, comunicação, reconhecer riscos, ter senso crítico para analisar informações conflitantes e tomar decisões relacionadas à saúde, que precisam ser considerados no planejamento e execução das ações em saúde^(22,23)^.

Na presente pesquisa, a análise de associação revelou significância estatística entre o SAHLPA-18 e o item “Calmo(a) e tranquilo(a)” do WHO-5, uma vez que a maioria dos participantes com LFS adequado respondeu estar mais da metade do tempo ou o tempo todo “calmo(a) e tranquilo(a)”. Este dado deve ser analisado à luz da importância do LFS para a manutenção de aspectos da qualidade de vida. A literatura mostra que o sentimento de bem-estar e qualidade de vida está ligado às relações familiares, de amizade e apoio social, além de estar relacionado com o grau de dependência funcional da pessoa^(24,25)^.

A ausência de significância estatística entre a percepção da pandemia da COVID-19 e o SAHLPA-18, demonstra que o nível de LFS não influenciou a percepção sobre a pandemia na amostra estudada. Apesar de alguns estudos evidenciarem que as medidas de distanciamento e proteção adotadas na pandemia tiveram impacto na saúde mental de parte da população idosa^(10,11,26)^, a literatura também mostra que as pessoas mais velhas tiveram melhores mecanismos para lidar o período de isolamento quando comparadas aos mais jovens^(10,11,27)^.

O enfrentamento de momentos de tensão e insegurança, como foi o caso da pandemia de Covid-19, requer recursos psicossociais e propostas resilientes no combate a crises. Na população idosa, isso se deu por meio da organização da rotina em casa e na comunidade, da melhoria do estilo de vida, incluindo a prática de atividade física; e do comportamento positivo, tendo como objetivo manter o bem-estar e apoiar socialmente as pessoas^(10,11,26,27)^.

Entre os resultados, foi observada correlação negativa de magnitude fraca entre o escore do SAHLPA-18 e os domínios Cognição e Autocuidado WHODAS 2.0. A pontuação do protocolo WHODAS é inversa, ou seja, quanto menor a pontuação, melhor o estado clínico funcional da pessoa, o que está em consonância com a literatura, que prevê que quanto melhor o letramento em saúde, melhores são as condições cognitivas e de autocuidado^(5,20)^.

Nesse contexto, vale destacar a importância de cuidadores e familiares para a população idosa com comprometimentos da cognição, não só para facilitar a gestão do cuidado, mas também para avaliar e consumir as informações em saúde. Da mesma forma que a consideração do LFS dos usuários se faz relevante para a efetividade do cuidado, os aspectos de letramento dos cuidadores também têm impacto na qualidade de vida destas pessoas^(28)^.

Estudos mostram que a falta de orientação e de suporte aos cuidadores geram exposição a estresse e sobrecarga, principalmente quando cuidam de sujeitos com maior grau de dependência. Associada à falta de prática de autocuidado das pessoas que cuidam, a condição de letramento em saúde influencia o desfecho da saúde daqueles sob seu cuidado. Aspectos como dificuldade de compreensão e interação com os profissionais de saúde, informações insuficientes ou dificuldade de avaliação da qualidade das informações, são exemplos de consequências do LFS inadequado que podem acometer os cuidadores e impactar o cuidado prestado^(28,29)^.

Como limitações do presente trabalho, vale citar que, por se tratar da etapa preliminar de um estudo observacional, o tamanho e o delineamento da amostra, não permitem generalizações. Além disso, pontua-se a possível restrição de alcance das perguntas relativas à percepção da pandemia de Covid-19.

Todavia, o desenvolvimento do estudo preliminar permitiu verificar a viabilidade da condução da pesquisa com uma amostra ampliada com estratificação por gênero, idade e aspectos funcionais para a obtenção de evidências mais robustas.

Um ponto fundamental do presente estudo foi a compreensão de que o letramento em saúde envolve múltiplas dimensões, e que a triangulação com as temáticas funcionalidade, qualidade de vida e percepção da felicidade mostra-se apropriada. É possível afirmar ainda que, a análise das associações do letramento em saúde com a percepção da pandemia pode contribuir para a discussão quanto à tomada de decisões das práticas de saúde e intervenções e adesão às intervenções em saúde pela população idosa.

## CONCLUSÃO

A análise de associação entre o LFS, aspectos sociodemográficos, funcionalidade, qualidade de vida, felicidade e percepção da pandemia pelas pessoas idosas revelou associação entre o LFS e o nível de escolaridade, a cognição, o autocuidado e se sentir calmo e tranquilo.

Portanto, pode-se afirmar que, na amostra estudada, as pessoas idosas que apresentaram melhor letramento em saúde, tiveram melhores capacidades cognitivas, fizeram melhor gestão do autocuidado, tiveram maior grau de instrução e apresentaram melhores resultados de bem-estar.
